# Effects of *Neospora caninum* infection on brain microvascular endothelial cells bioenergetics

**DOI:** 10.1186/1756-3305-6-24

**Published:** 2013-01-25

**Authors:** Hany M Elsheikha, Charlotte L McKinlay, Nashwa A Elsaied, Paul A Smith

**Affiliations:** 1 School of Veterinary Medicine and Science, Faculty of Medicine and Health Sciences, University of Nottingham, Sutton Bonington Campus, Leicestershire, LE12 5RD, UK; 2 School of Biomedical Science, University of Nottingham Medical School, Queens Medical Centre, Nottinghamshire, NG9 4BD, UK

**Keywords:** Adaptation, Blood–brain barrier, Trade-off, Mitochondrial respiratory chain, Oxygen consumption, *Neospora caninum*, Neuropathy

## Abstract

**Background:**

The brain is the most commonly affected organ during *Neospora caninum* infection but the mechanisms utilized by this protozoan parasite for traversal of the blood–brain barrier (BBB) are not yet understood. Herein, we investigate the cellular pathogenicity of *N. caninum* infection on bioenergetics of human brain microvascular endothelial cells (HBMECs), a fundamental component of the BBB.

**Methods:**

We tracked the growth kinetics of *N. caninum* in HBMECs. Focusing on cell bioenergetics, oxygen consumption rate (OCR) was determined using Clark electrode system and mitochondrial membrane potential (ΔΨm) was evaluated using DePsipher staining by fluorescence microscopy in the presence and absence of infection.

**Results:**

HBMECs provided a receptive environment for parasite proliferation. *N. caninum* tachyzoites were able to invade and replicate within HBMECs without significantly altering cell proliferation rate, as measured with the MTT assay, up to 24 hr post infection (pi). The oxygen consumption rate (OCR) was significantly inhibited (p < 0.001) by 10 mM glucose [from −2.26±0.23 to −0.6±0.21 nmol 10^6^ cell min^-1^ and from −0.29±0.09 to −0.16±0.1 nmol 10^6^ cell min^-1^ for uninfected HBMECs and free *N. Caninum* tachyzoites, respectively]. After normalization for DNA content the basal OCR did not differ between two host cell types: HBMECs and K562. The OCR of HBMECs was significantly elevated 24 hr pi in the absence of substrate, in 10 mM glucose and in the presence of a tetramethyl-p-phenylenediamine (TMPD)/ascorbate redox shuttle. Although quantitatively similar results were observed for uninfected K562 cells, there was no effect on their OCR 24 hr pi with *N. caninum* under any of the above substrate conditions. 6mM azide abolished OCR in all situations. Mitochondrial staining with DePsipher indicated no change in their membrane potential (Δψm) up to 24 hr pi.

**Conclusions:**

*N. caninum* is able to grow in HBMECs without markedly disrupting their normal proliferation or mitochondrial integrity. However, it is associated with an increase in infected cell respiration. Whether this increase reflects numeric addition of the parasites own respiration or results from an additional energy demand upon the host cell remains to be elucidated.

## Background

Infection with the obligate intracellular apicomplexan parasite *N. caninum* results in the disease neosporosis which produces clinical signs that are most evident in dogs and cattle [1,2]. Dogs with neosporosis can develop a wide range of neuromuscular defects including progressive hind limb ataxia or paresis and in severe cases, death [1,3]. In cattle, neosporosis commonly causes abortion or stillbirth [1,2]. However, *N. caninum* infection is generally latent and asymptomatic, and results in the formation of dormant encysted bradyzoites that remain in the brain and other tissues for life. Clinical signs in natural hosts, other than abortion, have only been reported sporadically in naturally congenitally-infected puppies and cattle younger than 2 months of age [1].

One of the important clinical consequences of *N. caninum* infection is the damage caused to the central nervous system (CNS), with the brain as the most commonly affected organ and is the site where most *N. caninum* cysts are found [1,4,5]. One of the key steps in the pathogenesis of cerebral neosporosis is *N. caninum* invasion of the CNS, which probably occurs at sites of the blood–brain barrier (BBB). The BBB is an active tissue [6], which under normal circumstances maintains tight regulation of substance entry to neuronal tissue [7], and the brain microvascular endothelial cells form a fundamental component of this barrier [8]. The ability of *N. caninum* to overcome this protective biophysical barrier is key to the ability of the parasite to damage the CNS [1,9].

Intracellular microbial agents rely on the host cells to provide energy and metabolic resources crucial for their survival and proliferation. As infection progresses, cellular stress increases, however, host cells have developed strategies to compensate metabolic shift as an attempt to maintain energy homeostasis and cell viability. Mitochondrial bioenergetics’ and metabolic balance are among the fundamental mechanisms used by cells to become tolerant to the stress caused by viral [10] or bacterial [11] infection. However, the extent to which parasites such as *N. caninum* alter host cell metabolism for their own replication, and the consequence of this disruption, remain unknown.

Considering the importance of understanding of the mechanisms underlying the neuropathogenicity of *N. caninum* and the lack of data about the interaction of this parasite with BBB cells, this study aimed to verify the susceptibility of brain microvascular endothelial cells to *N. caninum* infection and to elucidate the bioenergetic mechanisms used by these cells to adapt to *N. caninum* infection.

## Methods

### Cell lines

African Green Monkey kidney epithelial cells (Vero) were maintained in complete Dulbecco’s modified Eagle’s medium (cDMEM) supplemented with 10% (v/v) heat-inactivated fetal bovine serum (FBS), 2mM L-glutamine and 4% antibiotic-antimycotic mixture. Primary human brain microvascular endothelial cells (HBMECs) were used at passage 14 and were grown in complete RPMI-1640 Medium (cRPMI) supplemented with 20% heat inactivated FBS, 2mM L-glutamine, 1mM Sodium Pyruvate, 1% MEM non-essential amino acids, 1% MEM vitamins and 5ml penicillin/streptomycin. When cells were confluent they were harvested with trypsin-EDTA and passaged at a sub-cultivation ratio of 1:3 into new culture flasks with fresh corresponding medium (cDMEM for Vero cells and cRPMI for HBMECs). Cells were considered confluent when their expansion had reached a point where cells touched each other on all sides and no intercellular gaps could be observed. K562 cells, a human erythroleukemia cell were cultured in cRPMI medium supplemented with L-glutamine, 10% FBS and antibiotic–antimycotic mixture, and sub-cultured twice a week at initial concentration of 10^5^ cells ml^-1^. All cells lines were grown at 37°C under humidified 5% CO_2_ conditions. To exclude if cell viability could be regarded as a factor affecting parasite interaction with host cells and therefore any subsequent measurements, viability of cells was assessed on a minimum of 200 cells using the trypan blue exclusion assay prior to inoculation onto cultured flasks. Cell viability was greater than 95% at all times. Parasite viability was also checked using the Alamar Blue assay and only those greater than 95% were used. Vero cells and K562 cells were obtained from the European Collection of Animal Cell Cultures (Porton Down, Salisbury, Wiltshire, UK). All media, FBS, tissue culture supplements and antibiotics were from GIBCO. HBMECs were originally obtained from ScienCell Research Laboratories.

### Parasite culture

*Neospora caninum* (Nc-Liverpool) strain was obtained from Professor S. Trees (University of Liverpool) and was propagated in Vero cells as described [12]. Parasites were harvested from their feeder cell culture and purified as described previously [13]. The number of tachyzoites was estimated using a haemocytometer. The final volume of suspension was adjusted with culture medium to achieve a ratio of 1:1 parasite/host cell for subsequent infection experiments. Parasite viability was also checked using Alamar Blue assay and only those greater than 95% were used. All experiments were conducted at least in triplicate.

### *N. caninum* infection of HBMECs

HBMECs were grown on poly-L-lysine coated coverslips in 6-well cell culture plates and infected with *N. caninum* tachyzoites at a host-parasite ratio of 1:1. For controls, non-infected cells were sham inoculated with an equivalent volume of media without tachyzoites. After 1 hr of initial incubation, the media from the wells was removed and fresh cRPMI added. K562 cells were infected as described previously [12].

### Time-course of infection by *N. caninum*

To monitor the proliferation of tachyzoites within the host cell, cells were fixed in cold acetone:methanol (1:1) for 10 min at 1, 3, 6, 12, 24 and 48 hr post infection (pi). Infected cells and controls were processed for immunofluorescent staining as described [12]. Briefly, fixed cells were blocked in 10% FBS for 1 hr at ambient temperature and incubated with primary monoclonal mouse antibodies against anti-NcSAG1 (a kind gift from Professor Andrew Hemphill, University of Bern, Switzerland) in 1:400 dilution for 2 hr at ambient temperature. Alexa Fluor 488 (FITC filters) conjugated goat anti-mouse secondary antibodies (Invitrogen Ltd, Paisley, UK) at 1:500 dilution in TRIS buffered saline (TBS), were used to detect bound primary antibody by immunofluorescence after 2 hr incubation in the dark at ambient temperature. The coverslips were carefully removed from the cell culture plates and were mounted with ProLong Gold antifade reagent with 4′,6-diamino-2-phenylindole, dihydrochloride (DAPI; Molecular Probes, Inc., Eugene, OR) on microscopic slides. Images were captured on a Leica microscope (Leica Microsystems Imaging Solutions Ltd, Cambridge, UK). Because of the inherent subjectivity of the qualitative analysis, quantitative measurements of the surface area of the parasite were made by counting pixels or groups of pixels that were contributed by parasites. A region of interest from a large fluorescent image was selected and a threshold value for detection visually obtained from the green colour intensity of the parasites. The total number of pixels was counted automatically based on the green colour intensity above the threshold value. Using the scale bar on the image, the corresponding size and area of each pixel, and therefore, the surface area of parasites were calculated. The mean surface area ± standard deviation of the parasitic structure was measured per time points. Measurement of the parasite surface area was performed three times and are quoted in arbitrary units.

To assess the gross kinetics of *N. caninum* in HBMECs cells were cultured in 75cm^2^ culture flasks and grown in cRPMI. Once a confluent monolayer of cell was formed, parasites were added at a host-parasite ratio of 1:1. Flasks were viewed under the microscope every 24 hr and the number of parasitic lysis areas in 10 microscopic fields at x 10 magnification was recorded and the average for the flask was calculated. This experiment was repeated three times. Data is shown as average ± standard deviation.

### Detection of mitochondrial integrity and membrane potential (ΔΨm)

Mitochondrial integrity and membrane potential (ΔΨm) in normal and infected HBMECs was determined using the DePsipher kit (Assay Designs, USA) following the manufacturer’s instructions. This kit uses a unique cationic dye to indicate the loss of the mitochondrial potential. In cells with disrupted mitochondrial potential, the dye remains in the cytoplasm as a green fluorescent monomeric form, whereas in bioenergetic healthy cells the dye appears red following aggregation of the DePsipher dye within the mitochondria.

HBMECs were grown on coverslips as described above. Cells were infected with *N. caninum* tachyzoites at a host-parasite ratio of 1:1. Cells were stained at 1, 3, 6, 12, 24 and 48 hr pi. At each time point, the medium was discarded and cells were washed with 1ml pre-warmed (37°C) 1X diluted reaction buffer with stabilizer solution. Supernatant from diluted DePsipher solution centrifuged at 13,000 x g for 1 min at ambient temperature was added to the cells and they were then incubated for 30 min. Cells were washed again with 1ml pre-warmed 1X reaction buffer with stabilizer. The coverslip was then mounted onto a microscopic slide and viewed under the microscope immediately. The green monomers (Ex_485nm_/Em/_535nm_) and red aggregates (Ex_560nm_/Em_595nm_, a sensitive marker of ΔΨm) were monitored by fluorescence microscopy. Mitochondrial staining was repeated three independent times.

### Cell metabolism assays

To assess changes in host cell viability and bioenergetics following *N. caninum* infection, two different methods were employed. First, the colorimetric MTT assay [CellTiter 96 Non-Radioactive Cell Proliferation Assay; Promega, UK], which is based upon formazan production due to reduction by cellular reducing equivalents, was used. Although the MTT assay is usually utilized to monitor cell-proliferation, the fact that it depends on the cellular redox state means that it is essentially a measurement of total biological reduction potential within the assay volume [14]. The MTT assay was performed following the manufacturer’s instructions. Briefly, HBMECs were seeded in 96-well culture plates at 10^4^ cells per well and grown in cRPMI at 37°C in a humidified atmosphere containing 5% CO_2_. When the cells were confluent, tachyzoites of *N. caninum* were added at a host-parasite ratio of 1:1. Non-infected controls were sham inoculated with an equal volume of medium without tachyzoites. After 1 hr of initial incubation, medium from the wells was removed and fresh cRPMI was added. At 1, 2, 3, 6, 12, and 24 hr pi, 15μl of MTT dye solution was added to each well. Following incubation for 4 hr, 100μl of solubilisation solution/stop mix was added and incubated for a further 1 hr. Absorbance at 590 nm was recorded using a Multiskan Ascent plate reader [Labsystems]. This experiment was repeated at least 3 times.

The second method to assay changes in cellular bioenergetics was by measurement of the oxygen consumption rate [OCR] which results directly from mitochondrial oxidative respiration as previously described [15]. Briefly, the OCR of suspensions of free parasites, suspensions of infected or non-infected cell monolayers was measured polarographically using Clark Oxygen Electrodes [Rank Brothers, Bottisham, UK]. Suspensions of cells, volume 1 ml, were continuously stirred in sealed electrode chambers at 37°C. Since this is a closed system, the O_2_ consumed during oxidative respiration cannot be replaced from the air so the concentration of dissolved O_2_, [O_2__aq_ falls. [O_2__aq_ was measured at a polarographic voltage of −0.6 V. Electrodes were calibrated with a two point method in accord with the manufacturer’s instructions: 100% saturation with air [[O_2_]_aq_ ≈ 0.25 mM at 37°C] and 0% O_2_ by the addition of a stoichiometric excess of Na_2_S_2_O_4_, a strong reducing agent. These experiments were performed in a HEPES-buffered Hanks solution which contained (in mM): 138 NaCl, 4.2 NaHCO_3_, 1.2 NaH_2_PO_4_, 5.6 KCl, 1.2 MgCl_2_, 2.6 CaCl_2_, 10 HEPES (pH 7.4 with NaOH) and 0.1% fatty acid free BSA. Since the steady-state rates of oxygen consumption [OCR] were linear, OCR was taken as the slope in [O_2__aq_ over 3–5 min epoch. After a 10 min equilibration period, the OCR was measured first in the absence of exogenous substrate (0 glucose), then in the presence of 10mM glucose (by addition of 10μl of a 1M glucose stock solution in H_2_O to the 1ml incubation volume), then in the continued presence of 10mM glucose but with the addition of the electron donor shuttle pair: 0.2 mM tetramethyl-p-phenylenediamine with 1 mM ascorbate TMPD; [16]. To determine the OCR that arose solely through oxidative respiration, the mitochondrial reduction of O_2_ was then blocked by the addition of 6 mM NaN_3_ to abolish cytochrome c oxidase activity. To determine whether the cells, naïve or infected, or the free parasites themselves, preferentially metabolised monocarboxylic acids compared to glucose, their ability to respire L-lactate and methyl-pyruvate, a membrane permeable analogue of pyruvate, was tested. OCR was measured as described earlier, but with glucose substituted for by an equimolar amount of either L-lactate (by addition of 10μl from a 1M L-NaLactate stock solution, made in H_2_O, to the 1ml incubation volume) or methyl-pyruvate, a membrane permeable analogue of pyruvate (again by addition of 10μl from a freshly made 1M stock solution, made with H_2_O, to the incubation volume). Since lactate was without effect, no control was deemed necessary for the associated change in Na^+^ concentration: 138 to 148mM that occurred on addition of the lactate salt.

To control for O_2_ consumption that was either due to the background consumption by the electrode, which was non-mitochondrial in origin, or which arose due to autoxidation of ascorbate by atmospheric oxygen, all OCRs were corrected by subtraction of the OCR measured under identical experimental conditions in the absence of biological tissue or/and after the addition of NaN_3_. Each separate experiment constituted a biological replicate, and contributes to the experimental number: n.

Given the difference in cellular scale between tachyzoites and HBMECs, in order to compare OCR between the two different host cell types and free parasites we attempted to scale them by the ratio of their DNA content. It is well documented that the DNA content of cells at least in vertebrates is highly correlated and proportional to cell size [17]. It was assumed that this assumption also held true for the apicomplexan protozoa even though they have extra-nuclear DNA that is non-mitochondrial in origin. Normalization of OCR with DNA content does, however, allow for subsequent comparison to further studies and the literature. Total genomic DNA was extracted from 1 × 10^6^ of each of *N. caninum* tachyzoites, HBMECs or K562 cells, of similar density, using DNeasy Tissue kit (Qiagen, Valencia, California) following the manufacturer’s instructions. The extracted DNA was quantified by using a Nanodrop spectrophotometer (Nanodrop Technologies, Wilmington, DE). The ratio of DNA content relative to that in free tachyzoites was 6.44 for HBMECs and 8.6 for K562 cells; the DNA content being 10.3 pg μl^-1^ for HBMECs, 13.8 pg μl^-1^ for K562 cells, and 1.6 pg μl^-1^ for the tachyzoites.

### Statistical analyses

All data was checked for normality using the D’Agostino & Pearson omnibus normality test. Data is presented and analysed in a form appropriate to its distribution, with the test used stated in brackets. Parametric statistical analysis using PRISM (5.03, GraphPad Software, San Diego California, USA) were performed if data was deemed to be normally distributed, otherwise non-parametric analyses were performed with StatsDirect (StatsDirect Ltd, Altrincham, Cheshire, UK). Tukey’s Multiple Comparison Test was used for multiple comparisons across groups. When non-parametric data was compared with Kruskal-Wallis Conver-Inman [18], all groups were compared to each other, with only the specific comparisons quoted. Since the effects of glucose and TMPD/ascorbate within a given data group, as identified by host cell-type and state of infection (non-infected, 1 hr or 24 hr p.i.), were repeated measurements, the Friedman test was performed with all pairwise comparisons according to Conover [18]. All P values were two-tailed and adjusted for multiple comparisons when necessary using Bonferonni correction. P < 0.05 was considered statistically significant. The statistical test used, the P obtained when significant and n, the number of data determinations are given in parentheses.

## Results

### Parasite growth kinetics within HBMECs

**Figure 1:**
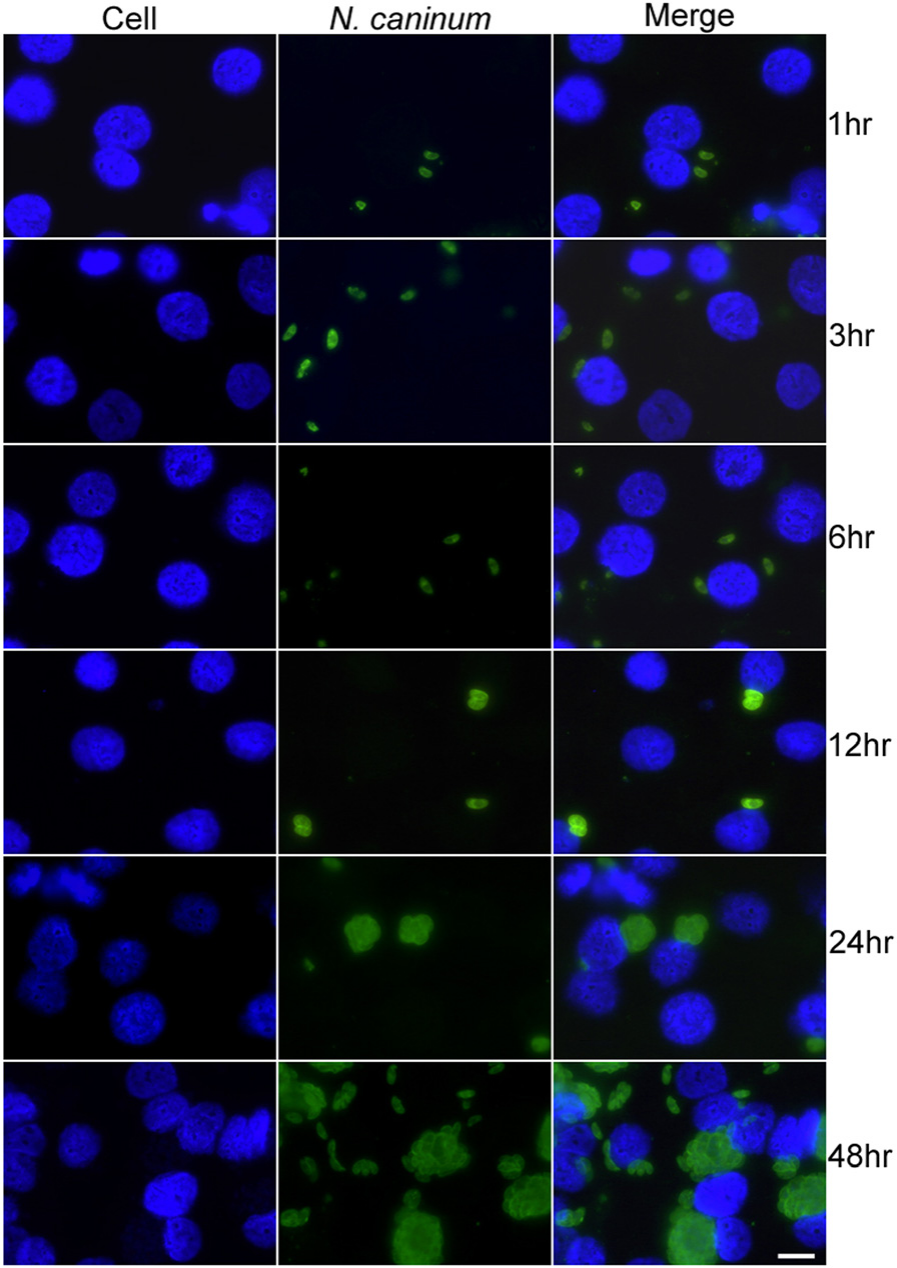
**Immunofluorescence staining of human brain microvascular endothelial cells [HBMECs] showing progressive increase in the number of *Neospora caninum *tachyzoites over time**. Cell nuclei [blue] and *N. caninum* tachyzoites [green]. Staining was performed as described in the methods at various time points post infection as indicated in the figure. (Scale bar = 20 *μ*l.).

Immunofluorescence staining and quantification of the surface area of the *N. caninum* tachyzoites infecting HBMECs enabled monitoring of the proliferation of tachyzoites during the first 48 hr pi (Figure 1). Between 1hr and 6hr pi of cells, tachyzoites increased in size from 38.7 ± 0.6 (1 hr, n = 3) to 59.1 ± 6.3 (6 hr, n = 3). By 12 hr pi, tachyzoites were beginning to divide by endodyogeny, as indicated by the increase in the tachyzoite’s surface area (123.5 ± 6.2, n = 3) and by 24 hr pi, multiple divisions had occurred (Figure 1) and large collections of tachyzoites were visible as spherical structures (surface area = 399 ± 22.5, n = 3). Over the next 24 hr period, more divisions took place and by 48 hr pi larger collections of dividing tachyzoites were visible (surface area = 1201 ± 87.8, n = 3). At 48 hr pi, some replications were complete, because the early tachyzoite stage of new replications were visible and were beginning to infect neighbouring cells.

Lysis areas appeared within the HBMECs by day 3 pi and extensive lysis areas throughout the flask were observed at 3–4 days pi, with free extracellular tachyzoites visible in the periphery of the lysis areas. Complete cell lysis occurred on day 6 pi.

### Effects on HBMEC metabolism

**Figure 2:**
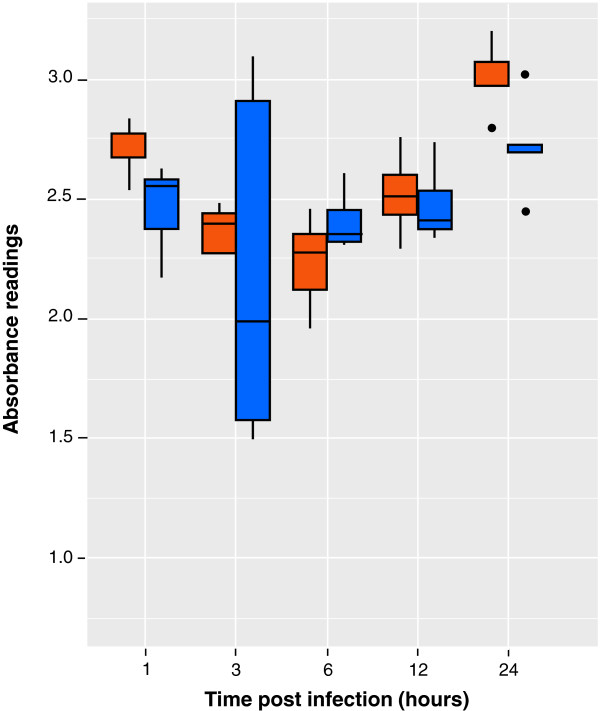
**The proliferation rate of human brain microvascular endothelial cells [HBMECs] infected and non-infected with *Neospora caninum *as a function of time in hours [x axis].** The proliferation of HBMECs with and without infection was assessed by the MTT assay. Group: Red = control HBMECs; blue = infected HBMECs. Data points are the mean of quadruplet readings. The intercept of the model was the absorbance at t = 1 hr in the control group. The model coefficients represent the difference to this reference. After having accounted for time, there was no significant difference between the control and infected group. Experiment was repeated at least 3 times.

Figure 2 shows that over the first 24 hr period of incubation there was no significant difference in the MTT assay for infected cells compared to control (P > 0.05; Tukey’s Multiple Comparison Test). However, the non-infected group of cells demonstrated a significant, but weak (r = 0.58, 95% confidence intervals 0.26 to 0.8) positive correlation of metabolic activity with time over the first 24 hr period (p < 0.01, Pearson Correlation). An observation that is consistent with the growth of the cells. Although a similar positive trend is suggested for the infected cells, this was just deemed insignificant (p = 0.071, Pearson).

**Figure 3:**
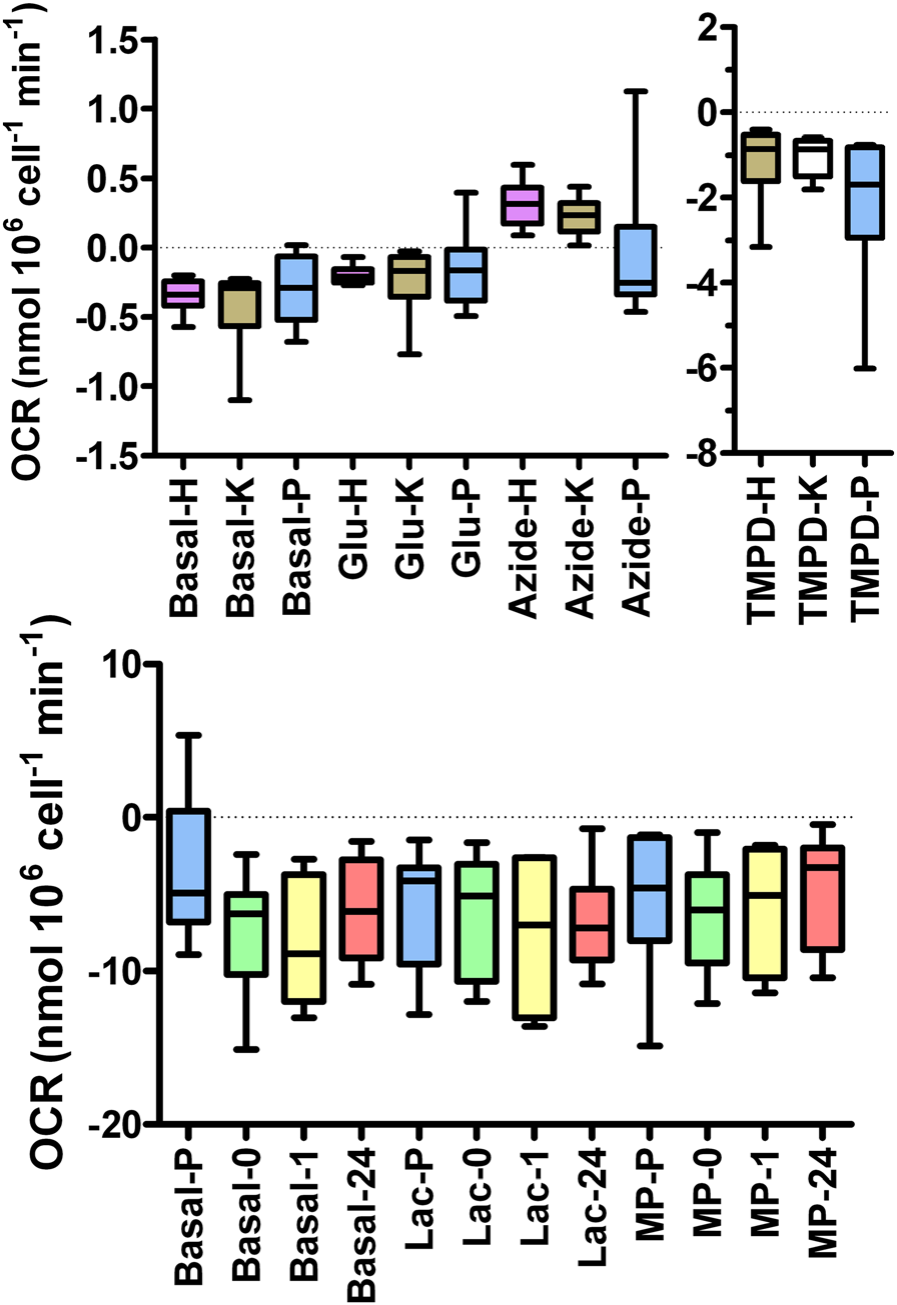
**Oxygen consumption rates (OCR) of uninfected cells, infected cells and free parasites preparations.** The rate of aerobic respiration was determined by the rate of O_2_ disappearance from an enclosed suspension of biological material. Since differences in the OCR of the tissues may arise due to differences of cell size and associated metabolic demand, the OCR for the host cells are normalised by the ratio of their DNA content to that of the parasite. **(Top panel)** Comparison of the OCR for uninfected HBMEC cells (H suffix, n = 10), and K562 cells (K suffix, n = 10) and free parasites (P suffix, n = 8) with various exogenous metabolic substrates as indicated. Key: Basal, no substrate; Glu, 10 mM D-glucose; TMPD, 10 mM glucose + TMPD/ascorbate redox couple, Azide as for TMPD + 6mM azide. **(Bottom Panel)** Comparisons of the OCR for free parasites (P suffix, n = 14), uninfected K562 cells (0 suffix, n = 16), infected cells 1 hr p.i. (1 suffix, n = 8) and infected cells 24 hr p.i. (24 suffix, n = 16) with various exogenous metabolic substrates as shown (n = 8 in all cases). Key: Basal, no substrate; Lac, 10mM D-lactate; MP 10 mM methyl pyruvate. Data in both panels is represented by box and whisker plots where: box, 25% to 75% quartile; bar, 50% quartile (median); whiskers, minimum and maximum.

Figure 3a shows that after correction for DNA content, the OCR of parasites was insignificantly different to that of uninfected HBMEC or K562 cells in each of the three substrate condition groups tested: basal, 10mM glucose or TMPD/ascorbate redox shuttle (Kruskal-Wallis Conver-Inman). In the absence of exogenous substrate, both HBMEC and K562 cells possessed an obvious measurable OCR, which was significantly inhibited by 40±10% [from −2.26±0.23 to −0.6±0.21 nmol 10^6^ cell min^-1^; n = 10; p < 0.02] and 50±8% [from −3.9±0.93 to −2.2±0.72 nmol 10^6^ cell min^-1^; n = 10; p < 0.01] respectively after addition of 10 mM glucose (Kruskal-Wallis Conver-Inman; Figure 3a). Subsequent addition of the mitochondrial electron transport chain electron donor couple, TMPD/ascorbate, resulted in a marked and significant stimulation of the OCR by 670±160% (n = 10; p < 0.001) and 820±330% (n = 8; p < 0.001) for the HBMECs and K562 cells respectively (Krusak-Wallis Conver-Inman; Figure 3a). Although the free parasites had similar, DNA corrected, values of OCR to the mammalian cell lines under the various substrate conditions, only addition of the TMPD/ascorbate redox shuttle produced a significant change in their OCR relative to their respective basal value (p < 0.001, Kruskal-Wallis Conver-Inman; Figure 3a). The cyanide equivalent, azide (6mM) which blocks cytochrome c of complex IV of the mitochondrial respiratory chain, under all substrate conditions for all cell systems studied, abolished OCR.

The glucose metabolism of K562, like other neoplastic tumour cell lines, is well established to predominantly glycolytic [19] as a consequence they are expected to have high lactate content. To determine if the tachyzoites had a preference for monocarboxylic acids over glucose as a metabolic substrate, hence favouring infection of this cell type, their ability to respire L-lactate and methyl-pyruvate, a membrane permeable analogue of pyruvate, were also tested. The OCR of uninfected cells, infected cells and isolated tachyzoites was unaffected by either 10mM D-lactate or 10mM methylpyruvate (Figure 3b).

**Figure 4:**
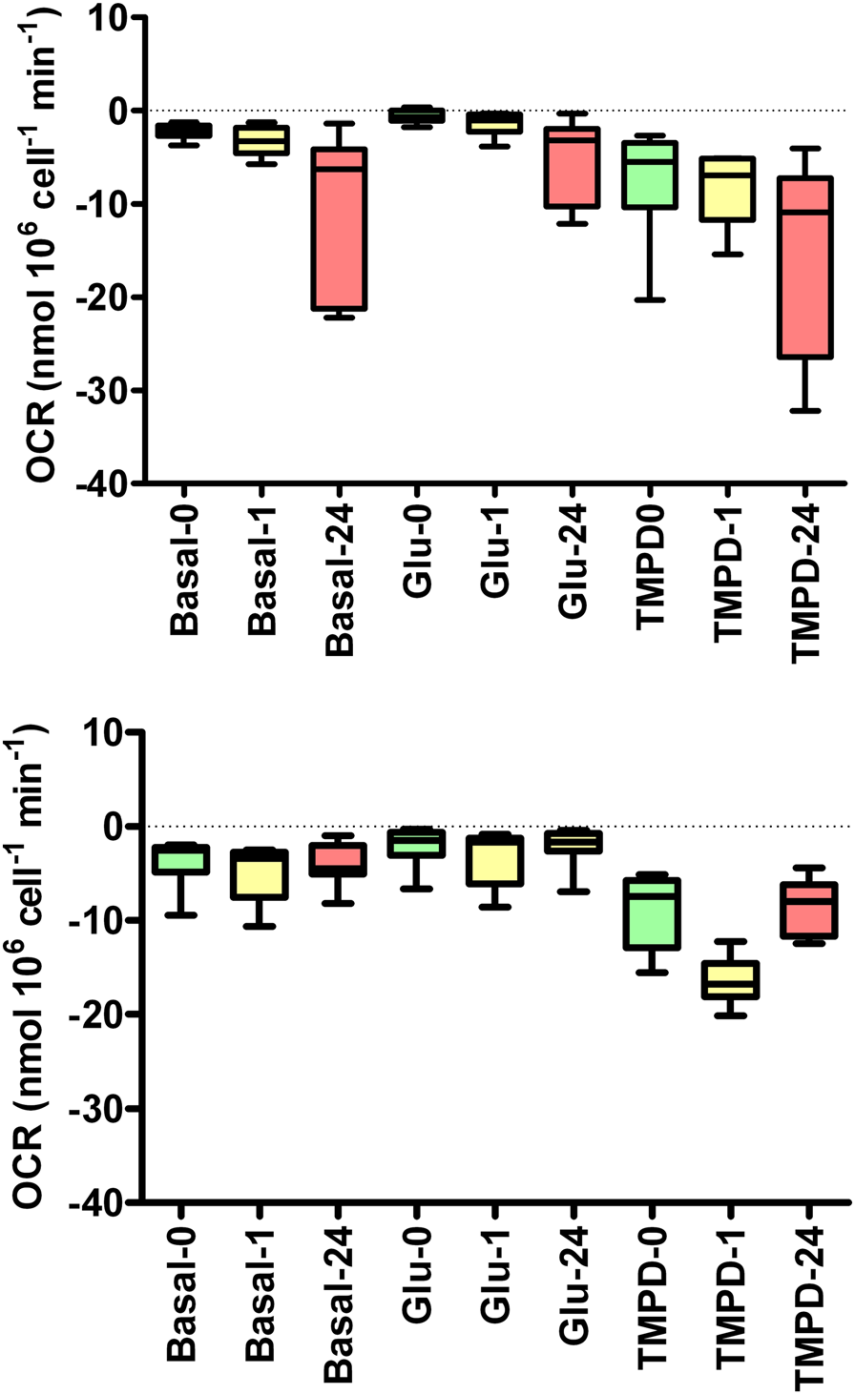
**Oxygen consumption rates (OCR) of uninfected cells, infected cells and free parasites preparations. (Top panel)** Comparison of OCR for uninfected HBMEC cells (0 suffix, n =10), HBMEC cells 1 hr post infection (1 suffix, n = 8) and HBMEC cells 24 hr post infection (24 suffix, n = 6) with various exogenous metabolic substrates. Key: Basal, no substrate; Glu, 10 mM D-glucose; TMPD, 10 mM glucose + TMPD/ascorbate redox couple. **(Bottom Panel)** Comparison of OCR for uninfected K562 cells (0 suffix, n =10), K562 cells 1 hr post infection (1 suffix, n = 8) and K562 cells 24 hr post infection (24 suffix, n = 10) with various exogenous metabolic substrates. Key as for top panel. Data is represented in the box and whisker plots as follows: box, 25% to 75% quartile; bar, 50% quartile (median); whiskers, minimum and maximum.

Figure 4a, shows that the OCR of HBMECs was significantly increased after 24 hr (P < 0.01) but not 1 hr, pi with *N. caninum* under all three substrate conditions (basal, glucose and TMPD/ascorbate) compared to their respective measurements in uninfected cells (Kruskal-Wallis Conver-Inman). The ability of 10 mM glucose to inhibit basal OCR was also still present in the HBMECs at both 1 hr (P < 0.05) and 24 hr pi (P < 0.05 Friedman test). Similarly, the ability of the TMPD/ascorbate electron donor couple to stimulate OCR in the HBMECs was also maintained post infection with *N. caninum* at both 1 hr (P < 0.05) and 24 hr (P < 0.05; Friedman test).

On the other hand, Figure 4b shows that the OCR of K652 cells was unaffected up to 24 hr pi with *N. caninum* either under basal or 10 mM glucose conditions (Kruskal-Wallis Conver-Inman). Although, like HBMECs, the K652 cells still possessed the ability for 10 mM glucose to inhibit their OCR at both 1 hr (P < 0.05) and 24 hr pi (P < 0.001; Friedman test). Contrary to what was observed in the HBMECs, the TMPD/ascorbate electron donor couple significantly stimulated OCR in the infected K652 cells in excess of that measured for the uninfected cells only at 1 hr (P < 0.05; Friedman test), but not 24 hr, pi. Moreover, the OCR at 24 hr pi in TMPD/ascorbate was almost identical to that measured in uninfected cells (Figure 4b).

### Effects on HBMEC proliferation and (ΔΨm)

**Figure 5:**
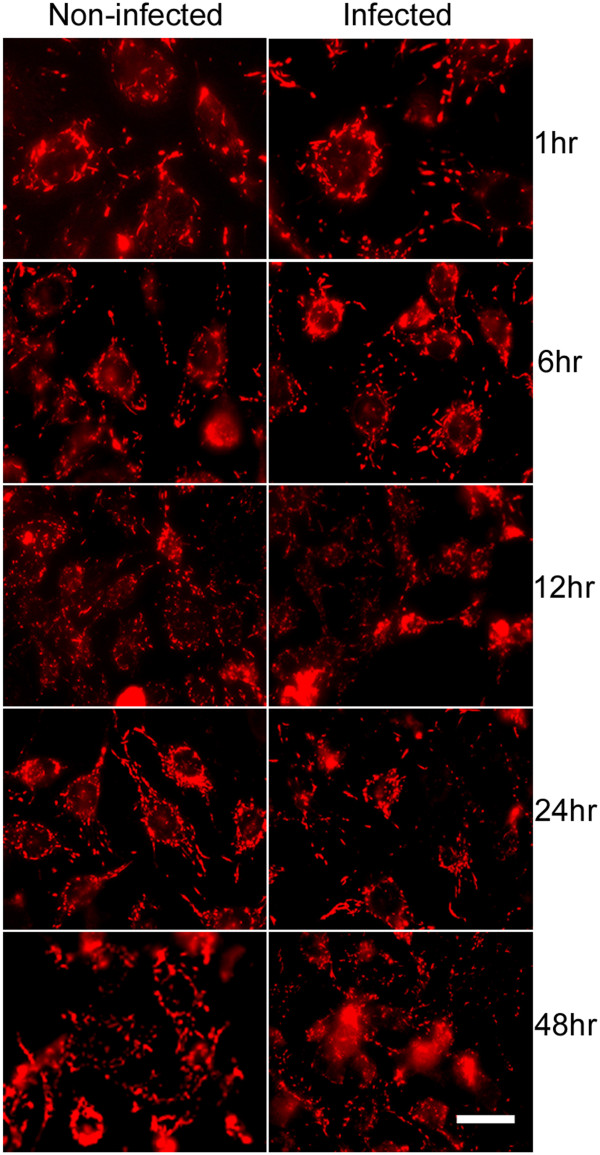
**Fluorescent mitochondrial staining with DePsipher kit.** No significant changes in the mitochondrial membrane potential [Δψm] of brain microvascular endothelial cells infected and non-infected with *Neospora caninum* over the time points indicated. (Scale bar = 20 *μ*l.)

As determined by DePsipher staining, which can serve as an indicator for mitochondrial membrane potential (Δψm) and activity, we did not observe any obvious reduction or disruption in Δψm following infection with *N. caninum* (Figure 5).

## Discussion

Given the lack of understanding of cerebral neosporosis, it is worthwhile to examine the host factors that mediate the complex interplay between *N. caninum* and the host cell microenvironment at the BBB interface using brain microvascular endothelial cells. First we analyzed the time course of infection. Since it was expected that most changes would occur early in infection we chose: 1, 3, 6, 12, 24 and 48 hr pi as the time points for the analysis. Infection of HBMECs with *N. caninum* was important for establishing whether normal invasion and proliferation occurs in these cells, and was a model of BBB. Immuno-fluorescence staining enabled visualization of *N. caninum* growth and confirmed that replication within HBMECs is possible by this parasite. *N. caninum* tachyzoites invaded and proliferated rapidly within the HBMECs and by 48 hr pi large numbers of replications had taken place and some tachyzoites were visibly spreading to infect neighbouring HBMECs. These findings indicate that HBMECs can support the growth and proliferation of *N. caninum*. The parasite exhibited a common pattern of growth and replication that has also been observed in cortical cells from rat brain infected with *N. caninum *[20]. Even though our data cannot be taken as conclusive evidence as a mechanism used by *N. caninum* for traversal of the BBB it provides a basis for further research on the traversal mechanisms used by *N. caninum* and the interactions that occur between *N. caninum* and host BMECs.

Utilization of an objective quantitative measurement of mean surface area of the parasite by computer aided digital image analysis has proved to be accurate and reproducible in observing and quantifying changes in the size of the growing parasite up to 48 hr pi.

The length of time for *N. caninum* tachyzoite proliferation within the HBMECs is similar to that found when organotypic slice cultures from rat cortical brain tissue were infected with *N. caninum*, where increased numbers of tachyzoites were observed around days 4 and 5 pi [20], which is consistent with the findings of our study. Profound infection was observed in rat cortical tissue by day 7 pi and this was accompanied by deterioration of the host tissue structure [20]. We made similar observations in the HBMECs by day 6 pi.

*N. caninum* is known to successfully infect a wide range of host cell types *in vitro *[1]. Our results demonstrated that HBMECs are readily infected by *N. caninum*, although presenting differences in susceptibility to infection compared to other cell lines. Bovine umbilical vein endothelial cells (BUVEC) have been also shown to support the propagation of *N. caninum* tachyzoites [21]. Parasite replication and subsequent release from ruptured UVECs occurred at 72 hr pi (ie, 3 days earlier than HBMECs).

The threat of zoonotic potential [22-24] has raised public health concerns about humans being a possible host of *N. caninum *[1]. Human cell lines have been used to verify the susceptibility of human trophoblastic (BeWo) compared with uterine cervical (Hela) cell lines to *N. caninum *[25]. Human uterine cells were found to use similar effector mechanisms to those described in bovine cells to control *N. caninum* infection. Thus, HBMECs can be considered in comparative approaches to understand potential strategies used by *N. caninum* to survive in human tissue. However, caution should be exercised when extrapolating results obtained in cells of human origin (HBMEC and K562) to cell types present in the natural hosts (bovine or canine), since their response to the infecting parasite can be different. Further research using bovine endothelial cells is needed. However, this study is a first step towards improving our understanding of the complex cellular events that occur during *N. caninum* replication in cell lines of non-natural hosts. Also, it offers insights into the nature of permissivity of new lines to *N. caninum* infection, thus provides useful information regarding cell adaptation and host specificity.

The MTT assay did not show a significant difference in the proliferation rate between *N. caninum*-infected and control HBMECs during the first 24 hr of infection; data which suggest that *N. caninum* is able to invade and replicate within HBMECs without causing substantial and notable cellular damage during the early infection process. A similar finding was obtained in primary cultures of rat astrocytes infected with *N. caninum*, where LDH levels increased significantly in culture supernatants after 24 hr and 72 hr of infection, compared with non-infected controls, but without significant changes in cell viability determined by MTT assays [20]. *N. caninum* infection of mouse embryonic fibroblasts has been found to result in reduced caspase activity and thus, inhibition of apoptosis [26]. The same process may be occurring during *N. caninum* infection of HBMECs.

The OCR of uninfected K562 cells we measured, -2.2±0.72 nmol 10^6^ cell min^-1^, was similar to that previously published for the same cell line: -3.2±0.56 nmol 10^6^ cell min^-1^[27]. This is the first time, however, that an OCR for HBMECs has been reported. Both cell types demonstrate a clearly significant Crabtree effect [28]: inhibition of basal OCR in response to an elevation of extracellular glucose. The Crabtree effect is commonly observed in cell lines; an effect thought to be due to feed forward inhibition of oxidative respiration by glycolytic products [29]. No previous data knowingly exists for the OCR of *N. caninum*. Interestingly however, the azide-sensitive, OCR of free tachyzoites in the absence of exogenous substrate we report (−0.16±0.1 nmol 10^6^ cell min^-1^) is quite similar to the cyanide–sensitive OCR found for the related apicomplexan, *Toxoplasma gondii* (−0.09±0.07 nmol 10^6^ cell min^-1^)[30]. The fact that the free parasites had an oxygen consumption in the absence of exogenous substrate demonstrate they possess a basal oxidative metabolism; possibly utilizing endogenous fat from the lipid-like inclusions described in this organism [31,32], whether they contain glycogen is unknown. Furthermore, the fact that the oxygen consumption of the free tachyzoites was abolished by azide suggests that they too have a singular respiratory pathway similar to *T. gondii *[33] and do not possess a branched respiratory pathway like that found in other members of the apicomplexan phylum e.g. *Plasmodium falciparum *[33,34].

The inability of glucose, lactate or methylpyruvate to stimulate OCR in any of the systems studied supports the notion that glycolytic flux in these organisms are tightly controlled; most likely occurring at the level of pyruvate dehydrogenase (PDH) whose modulation is considered the prime candidate for the Crabtree effect [29], a phenomenon we observed for both host and infected cells. Since we did not observe a Crabtree effect in the free tachyzoites, nor indeed a basal OCR affected by lactate or methylpyruvate, is evidence that strongly argues for an inactive, or even absent, glycolytic pathway \in this parasite. Such a notion has already been suggested for *T. gondii*, in which exogenous pyruvate failed to simulate oxidative respiration even when their plasma membrane was permeabilised by digitonin to allow this metabolite direct access to the mitochondria [33]. This latter data suggest the absence of various dehydrogenases in *T. gondii* and is a finding consistent with the lack of a Crabtree effect we noted for *N. caninum*. We found that the respiratory electron donor shuttle, TMPD/ascorbate, was capable of stimulating oxidative respiration in the free tachyzoites, an effect abolished an inhibitor of cytochrome c (azide); again findings consistent with those that previously observed for permeabilised *T. gondii *[33].

After 24 hr pi there was a clear enhancement of OCR in the HBMECs, but not K562 cells. Given that glucose almost abolished OCR in the uninfected HBMECs, but only had a partial Crabtree effect after 24 hr pi suggests that the remaining OCR in HBMECs in the presence of glucose may in fact be due to simple numerical addition of the respiration from the internalized parasites. From electron microscopy and light microscopy images of infected host cells, we estimate that there are between 40–100 parasites in HBMECs at this time point, which, on the basis of their respiration in the free state may account for −6 to −15 nmol min^-1^ 10^6^ cell^-1^ of the infected host cell OCR: values close to the OCR range, -2 to −10 nmol min^-1^ 10^6^ cell^-1^, actually measured in HBMECs 24 hr pi in the presence of 10 mM glucose when the oxidative respiration of the host cell but not parasite is presumably inhibited by the Crabtree effect. Alternatively, the elevation of host cell OCR may just represent a cellular response to infection. In contrast, we estimate that K562 cells contain between 15–40 parasites at 24 hr pi, which by simple addition would be expected to enhance the OCR of this host cell by between −3 to −8 nmol min^-1^ 10^6^ cell^-1^. However, such an enhancement was not observed, possibly because for this particular host cell type the respiration of the internalized parasite is suppressed or inactive. This notion is supported by the observation that *T. gondii* loses its mitochondrial membrane potential, Δψm, and by induction, its oxidative respiration, after invasion of certain cell types, for example 3T3 fibroblasts [35].

Disruption of cellular Δψm has been demonstrated to be a causative factor for the occurrence of necrosis and apoptosis, and is often associated with oxidative stress [36]. Mitochondrial staining showed that *N. caninum* did not compromise the mitochondrial Δψm of infected HBMECs up to 48 hr pi, a finding which also rules out apoptosis as a possible mechanism for the enhancement of OCR seen with infection. Similar observations were reported when rat astrocytes were infected with *N. caninum in vitro *[20]. Taken together, OCR findings corroborate with the findings of the MTT assay and further substantiate previous findings [26] namely that of the capacity of the parasite to manipulate the host cell in order to prevent damage to the host cell and to allow its normal functioning during the early stage of infection. Whilst further studies are necessary to disclose the molecular mechanisms underlying *N. caninum*-induced cellular death, the groundwork established in the present work suggests that cell respirometry analysis for the investigation of mitochondrial bioenergetics of brain microvascular endothelial cells, especially at the early stage of infection, may be an important tool for understanding *N. caninum*-host cells interactions.

## Conclusions

Our data indicate that intracellular growth and proliferation of *N. caninum* within HBMECs is possible without significantly disrupting the normal functioning of the host cell during the first 24 hr of infection or its mitochondrial Δψm during the first 48 hr of infection. These findings underscore the importance of assessing the biological reasons and implications of trade-offs between maintenance of a stressed (*N. caninum*-infected) cell and energy costs of fitness-related functions such as metabolism and growth in order to provide better insight into the dynamics of parasite replication and host cell survival. We argue that the parasite could modify the cellular environment by blocking cell death or the apoptotic pathway during early infection to promote its own intracellular survival. This work provides the basis for a better understanding of the complex host-*N. caninum* interaction and for further studies on the brain blood barrier cell bioenergetic response to *N. caninum* infection, preferentially of canine or bovine origin.

## Abbreviations

BeWo: Human trophoblastic cells; BBB: Blood–brain barrier; CNS: Central nervous system; DMEM: Dulbecco’s modified Eagle’s medium; HBMECs: Human brain microvascular endothelial cells; HeLa: Uterine cervical cells; RPMI: RPMI-1640 Medium; OCR: Oxygen consumption rate; PI: Post infection; TBS: Tris buffered saline.

## Competing interest

The authors declare that they have no competing interests.

## Authors’ contributions

HME and PAS conceived and designed the experiments. CLM, HME, PAS and NAE performed the experiments. PAS and HME analyzed the data. HME, PAS, and CLM contributed to drafting the manuscript. All authors have read and approved the final version of the manuscript.
